# Comparative Analysis of Inflammatory Indexes in Lung Cancer Patients With and Without Brain Metastases

**DOI:** 10.7759/cureus.60921

**Published:** 2024-05-23

**Authors:** Flaviu Tamas, Corina I Tamas, Bogdan A Suciu, Doina R Manu, Alina R Cehan, Adrian F Balasa

**Affiliations:** 1 Neurosurgery, George Emil Palade University of Medicine, Pharmacy, Science and Technology of Târgu Mureș, Târgu Mureș, ROU; 2 Neurosurgery, Emergency Clinical County Hospital, Târgu Mureș, Târgu Mureș, ROU; 3 Anatomy and Embryology, George Emil Palade University of Medicine, Pharmacy, Science and Technology of Târgu Mureș, Târgu Mureș, ROU; 4 Thoracic Surgery, Emergency Clinical County Hospital, Târgu Mureș, Târgu Mureș, ROU; 5 Biological Sciences, George Emil Palade University of Medicine, Pharmacy, Science and Technology of Târgu Mureș, Târgu Mureș, ROU; 6 Plastic and Reconstructive Surgery, Emergency Clinical County Hospital, Târgu Mureș, Târgu Mureș, ROU

**Keywords:** systemic immune-inflammation index (sii), platelet-to-lymphocyte ratio, lymphocyte-to-monocyte ratio, neutrophil-to-lymphocyte ratio, brain metastases, lung cancer

## Abstract

Introduction

Lung cancer is the leading cause of oncological deaths worldwide. Various combined inflammatory indexes, such as the systemic immune-inflammation index (SII), neutrophil-to-lymphocyte ratio (NLR), lymphocyte-to-monocyte ratio (LMR), and platelet-to-lymphocyte ratio (PLR) have shown associations with pretreatment survival prognosis in patients suffering of lung cancer with or without brain metastases. This study aimed to compare the average values of NLR, PLR, LMR, and SII in healthy patients, patients with lung cancer without any other metastases, and patients with lung cancer and brain metastases.

Materials and methods

In this prospective study, we have divided the patients into three groups: Group 1 included patients diagnosed with lung cancer and one or more brain metastases of lung cancer origin, Group 2 included patients diagnosed with lung cancer without known metastases, and Group 3 was the control group which included healthy subjects. Preoperative complete blood counts were extracted for all included patients and we calculated the values of SII, NLR, PLR, and LMR for each individual patient in each group. The next step was to calculate the average values of SII, NLR, PLR, and LMR for each group of patients and to identify the differences between groups.

Results

A total number of 228 patients were enrolled in the study. Group 1 included 67 patients with average values of SII = 2020.98, NLR = 7.25, PLR = 199.46, and LMR = 2.97. Group 2 included 88 patients with average values of SII = 1638.01, NLR = 4.58, PLR = 188.42, and LMR = 3.43. Group 3 included 73 subjects with the following average values of the inflammatory indexes: SII = 577.41, NLR = 2.34, PLR = 117.84, and LMR = 3.56.

Conclusion

We observed statistically significant differences in SII, NLR, and PLR among the three groups of patients, suggesting their potential role as prognostic markers. Furthermore, our analysis revealed significant correlations between inflammatory markers within lung cancer patients, highlighting their involvement in tumor microenvironment modulation. Our findings demonstrate an escalation in SII, NLR, and PLR values as the disease progresses. These parameters of inflammation and immune status are readily and cost-effectively, and repeatedly assessable in routine clinical practice.

## Introduction

Lung cancer (LC) is the leading cause of oncological deaths worldwide due to its initial lack of symptoms leading to its diagnosis in advanced stages. With an increasing rate in the last years, LC has a higher mortality rate than breast, prostate, and colon cancers altogether. Smoking along with an increased genetic susceptibility are the two main reasons for LC development, with women being less affected than men [1].

There are two types of LC: small cell lung carcinoma (SCLC) (accounting for 20%) and non-small cell lung carcinoma (NSCLC) (accounting for 80%), which is further subdivided into its three most common forms: adenocarcinoma, squamous cell carcinoma, and large cell carcinoma. Most LC deaths are caused by brain metastasis (BM). It has been shown that up to 50% of LC finally develop into BM and more than half of the total number of BM have their origin from LC [2].

Smokers who develop a new type of coughing, patients with recurrent pneumonic episodes within the same anatomic location, or patients who present frequent flare-ups of their chronic obstructive pulmonary disease may be suspects of LC. Other clinical manifestations may be dyspnea, hemoptysis, hoarseness, pleural or pericardial effusions, or pain in the thoracic area [1]. On the other hand, patients presenting with BMs may be initially asymptomatic or may present with signs of raised intracranial pressure like headaches, nausea, vomiting, depressed consciousness up to coma, and depending on the tumor location epileptic seizures, motor deficits, or aphasia may be present.

Early-stage LC can be diagnosed through positron emission tomography (PET) scans and in more advanced stages chest radiography, thoracic contrast-enhanced computer tomography (CECT), or magnetic resonance imaging (MRI) scans can reveal the diagnosis of the primary tumor. BMs may be diagnosed by CECT or MRI scans. Sampling may be performed through endobronchial ultrasound-guided bronchoscopic sampling and/or stereotactic brain biopsy if patients present with BMs, followed by histopathological confirmation and molecular testing, which is vital for choosing the best possible treatment option for each patient [3].

Treatment of NSCLC may consist of surgical resection, radiotherapy, chemotherapy, immunotherapy, and/or targeted therapy. The diagnosis of SCLC should be made using the least invasive method possible. SCLC patients are treated with systemic therapy and/or radiotherapy; surgery is indicated in less than 5% of the cases followed by postoperative chemotherapy. Cranial irradiation is recommended as prophylaxis if patients respond well to the initial treatment, regardless of the stage of the disease [1]. Patients with BMs may benefit from complete surgical resection of all the tumors if they are surgically accessible. In patients presenting with multiple metastases and one large metastasis located in a critical area such as the mesiotemporal area or the posterior fossa compressing the brain stem and endangering the patient's life, palliative resection may be considered. For patients with multiple BMs, some of which are surgically inaccessible, neuronavigation-guided stereotactic brain biopsy can be performed for diagnosis confirmation [4]. 

Various combined inflammatory indexes, such as the systemic immune-inflammation index (SII), neutrophil-to-lymphocyte ratio (NLR), lymphocyte-to-monocyte ratio (LMR), and platelet-to-lymphocyte ratio (PLR) derived from counts of peripheral lymphocytes, neutrophils, monocytes, and platelets have shown associations with pretreatment survival prognosis in patients suffering of LC with or without BMs [5-7]. These inflammation and immune status parameters are easily accessible, cost-effective, and repeatedly assessable during routine clinical practice. However, to our knowledge, a direct comparison of the average values of these systemic immune and inflammatory indexes, including SII, NLR, PLR, and LMR, hasn't been performed between healthy patients, patients suffering from LC without known metastases, and patients presenting with BMs of LC origin. Thus, this study is aimed to inform future therapeutic development by comparing the systemic immune-inflammatory status of healthy patients, presurgical patients with LC without any other metastases, and patients with LC BMs needing neurosurgical resection by analyzing how these values change in different stages of the disease and identifying any statistically significant differences between the average values of the SII, NLR, PLR, and LMR between these three groups of patients. 

## Materials and methods

This was a prospective study that included patients admitted to the Neurosurgery and Thoracic Surgery Departments of the Emergency Clinical County Hospital, Târgu Mureș, Romania, between December 2020 and December 2023. The study was approved by the Medical Ethics Commission for Clinical Medicine of the Emergency Clinical County Hospital of Târgu Mureș (approval number: Ad. 30292).

Inclusion and exclusion criteria

Inclusion Criteria

Patients with complete records, Karnofsky performance status (KPS) > 70, no steroid medication before blood sampling, no history of radiation therapy or chemotherapy, no acute or chronic infections, no history of hematological diseases, complete imaging records, histopathological confirmation of LC or LC origin of BM were included in Groups 1 and 2. Healthy subjects were included as controls in Group 3.

Exclusion Criteria

Patients suspected or known with other malignant tumors, patients presenting with lung metastases located elsewhere except the brain and lungs, patients who received steroid medication, anti-inflammatory or immunosuppressive drugs before the initial blood sampling, patients who received radiotherapy, patients suffering from chronic or acute infections, patients with hematological or autoimmune diseases, patients with skull metastases or leptomeningeal carcinoma (metastasis), and patients with incomplete or missing records were excluded.

Participants

The participants were divided into three groups: Group 1 included patients diagnosed with LC and at least one BM of LC origin, Group 2 included patients diagnosed with localized LC without known metastases, and Group 3 was the control group which included healthy subjects (volunteers). All the included patients from the first two groups underwent surgical tumoral resection or biopsy. From our database, we extracted the preoperative complete blood counts for each of the included patients. As a standard procedure in our hospital, blood samples are collected on fasting patients during the admitting morning. From the patients' records, we confirmed that they were not on steroid medication before blood sampling. There were no notable distinctions in age or gender between groups.

Data collection

Data including age, sex, histopathological diagnosis, number of BMs, and routine laboratory variables including platelets (P), neutrophils (N), lymphocytes (L), and monocytes (M) were obtained and analyzed for each patient. All patients included in this study had KPS over 70. The SII, NLR, PLR, and LMR values were calculated for each patient using the following formulas: SII = P * N/L; NLR = N/L; PLR = P/L and LMR = L/M. The average values of the SII, NLR, PLR, and LMR were then calculated for each of the three groups. The first two oncological groups were diagnosed using preoperative contrast-enhanced brain MRI and preoperative thoracic/abdomen/pelvis CECT scans which were analyzed by our senior authors (AB and BS) and an independent radiologist. The diagnosis was then confirmed by the postoperative histopathological exam.

Statistical analysis

The frequency tables, graphics, and statistical tests were performed using IBM SPSS Statistics for Windows, Version 29.0 (Released 2022; IBM Corp., Armonk, New York, United States). Quantitative variables were checked for normal data distribution using the Kolmogorov-Smirnov test (with Lillefors correction). Means were reported along with standard deviations (SD) with a 95% confidence interval (CI). To evaluate the homogeneity of variances, Levene's test was performed, followed by the ANOVA test. To confirm the ANOVA test results were real and not incidentally obtained we have performed the ANOVA Effect Sizes test. Post Hoc Tests (Tukey) were performed to identify which of the three groups presented differences. Regarding the qualitative variables, the frequencies were calculated, and the related graphs were made. Correlations were also tested using the Pearson correlation coefficient. In all these cases, differences were statistically significant at a threshold value (p) of less than 0.05.

## Results

A total number of 228 patients fulfilled the inclusion criteria and were enrolled in this study. Table 1 summarizes the clinicopathological parameters of the included patients.

**Table 1:** Clinicopathological parameters of the patients included in this study (N=228) KPS: Karnofski performance status; SCLC: small cell lung cancer; NSCLC: non-small cell lung cancer; SII: systemic immune-inflammation index; NLR: neutrophil-to-lymphocyte ratio; LMR: lymphocyte-to-monocyte ratio; PLR: platelet-to-lymphocyte ratio; BM: brain metastasis

	Group 1 (N=67)	Group 2 (N=88)	Group 3 (N=73)	p-value
KPS	> 70	> 70	> 70	
Age (years), mean (range), SD	64.5 (34-80), SD=9.87	63.69 (43-81), SD=7.89	63.08 (45-82), SD=10.57	
Males, n (%)	41 (61.1%)	62 (70.5%)	38 (52.1%)	
Females, n (%)	26 (38.8%)	26 (29.5%)	35 (47.9%)	
SCLC, n (%)	6 (8.9%)	6 (6.9%)		
NSCLC, n (%)	61 (91.04%)	82 (93.1%)		
SII	2020.98	1638.01	577.41	p < 0.001
NLR	7.25	4.58	2.34	p < 0.001
PLR	199.46	188.42	117.84	p < 0.001
LMR	2.97	3.43	3.56	p > 0.05
BM, average number	2.22/patient (min 1/patient max 11/patient), SD = 2.15			p > 0.05
BM, average volume	19.35 ml (min 1.05ml max 77 ml), SD = 20.09			p > 0.05

Group 1 included 67 patients (41 males and 26 females) with a mean age of 64.5 years (range 34-80, SD=9.87) with newly diagnosed BMs of LC origin. The number of BMs ranged between one to 11 metastases/patient, with an average value of 2.22 metastases/patient (SD=2.15), and the average BM tumoral volume was 19.35 ml ranging between 1.05 ml and 77 ml (SD = 20.09). We did not find any statistically significant correlations between the inflammatory markers and the number and volume of BMs (p > 0.05). Regarding localization, most of the metastases were in the right frontal lobe (15.7%) followed by the left frontal lobe (11.4%), and 91.04% of the metastases were of NSCLC. The average values of the inflammatory indexes in this group were: SII =2020.98, NLR = 7.25, PLR = 199.46, and LMR = 2.97. 

Group 2 included 88 patients (62 males and 26 females) with newly diagnosed LC without any diagnosed metastases. The average age in this group was 63.6 years (range 43-81 years, SD = 7.89). Similar to Group 1, most of the patients in Group 2 had NSCLC (93.1%), and only 6.9% of the patients were diagnosed with SCLC. Regarding localization, 12.1% of cancers were in the right superior lobe followed by the left superior lobe (9,9%). The average values of the inflammatory indexes in this group were: SII = 1638.01, NLR = 4.58, PLR = 188.42, and LMR = 3.43.

Group 3, the control group, included 73 patients (38 males and 35 females) with an average age of 63.08 years (range 45-82 years, SD = 10.57). The average values of the inflammatory indexes in this group were: SII = 577.41, NLR = 2.34, PLR = 117.84, and LMR = 3.56.

**Figure 1:**
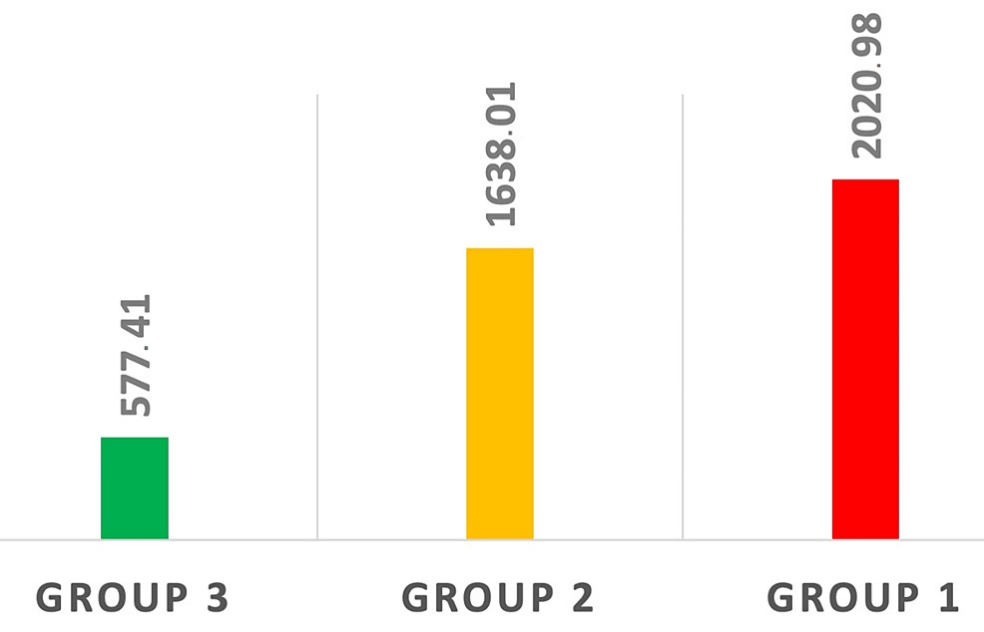
Modification in SII among the three groups. Statistically significant differences were observed between healthy patients (Group 3), patients with localized LC (Group 2), and patients with LC BM (Group 1). p < 0.05 SII: systemic immune-inflammation index; LC: lung cancer; BM: brain metastasis

**Figure 2:**
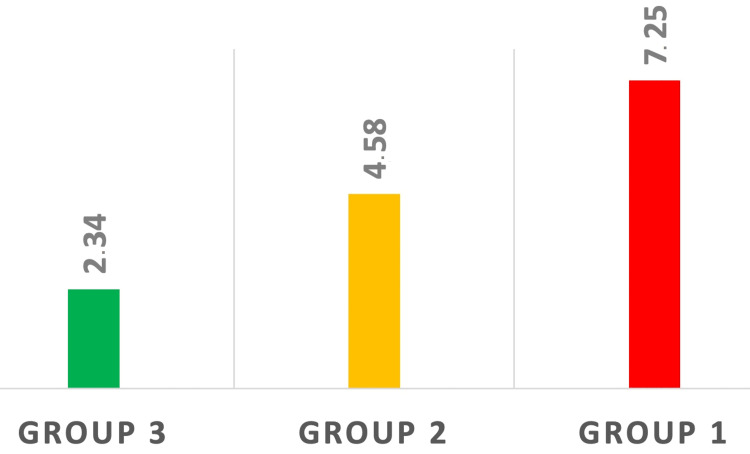
Modification in NLR among the three groups. Statistically significant differences were observed between healthy patients (Group 3), patients with localized LC (Group 2), and patients with LC BM (Group 1). p < 0.05 NLR: neutrophil-to-lymphocyte ratio; LC: lung cancer; BM: brain metastasis

**Figure 3:**
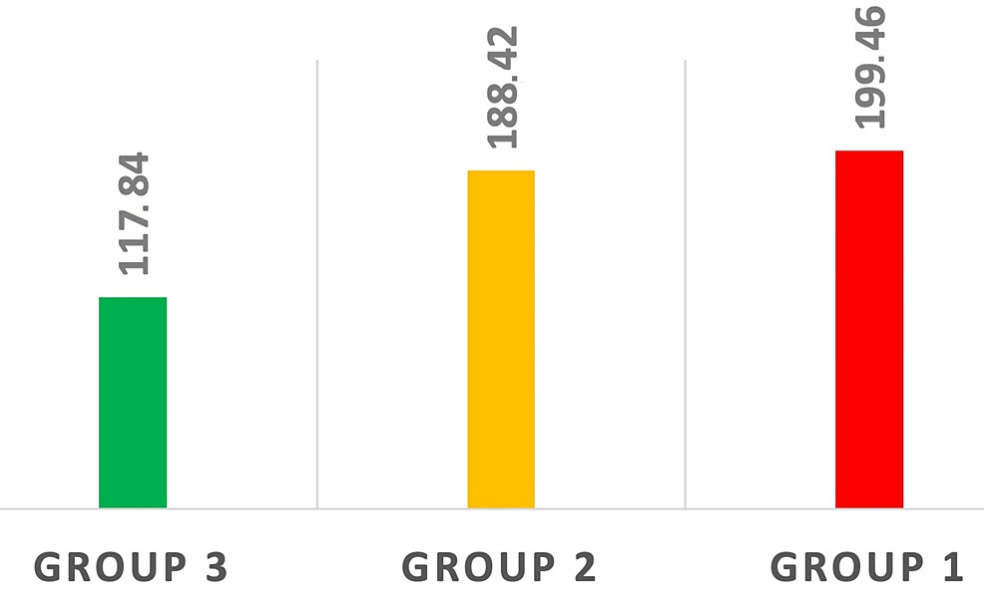
Modification in PLR among the three groups. Statistically significant differences were observed between healthy patients (Group 3), patients with localized LC (Group 2), and patients with LC BM (Group 1). p < 0.05 PLR: platelet-to-lymphocyte ratio; LC: lung cancer; BM: brain metastasis

High statistically significant differences in the mean SII, NLR, and PLR could be seen between the three groups of patients after ANOVA testing (p < 0.001) (Figures 1-3).

**Figure 4:**
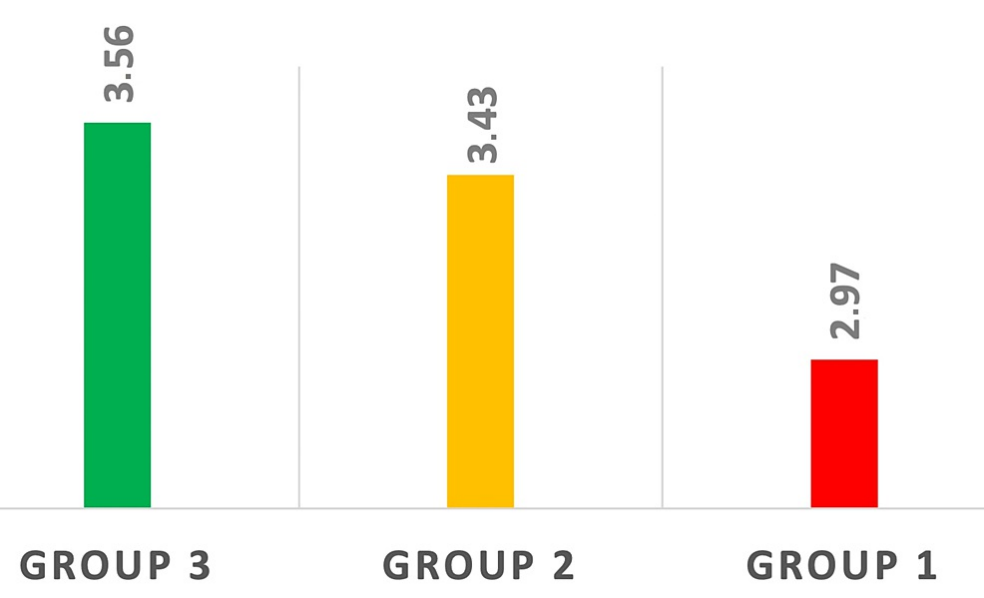
Difference in mean LMR values between the three groups of patients. These differences were not statistically significant. p > 0.05 LMR: lymphocyte-to-monocyte ratio

The differences in the mean values of LMR were not statistically significant (p > 0.05) (Figure 4).

The ANOVA Effect Sizes test resulted in SII small effect (epsilon-squared point estimate of 0.053, 95% CI), NLR large effect (epsilon-squared point estimate of 0.116, 95% CI), PLR medium effect (epsilon-squared point estimate of 0.102, 95% CI), and LMR small effect (epsilon-squared point estimate of 0.006, 95% CI).

After the post hoc test (Tukey), high statistically significant differences were obtained in the mean SII value between Group 3 and Group 1 (mean difference = - 1443.57, p < 0.001), NLR value between Group 3 and Group 1 (mean difference = - 4.91, p < 0.001), PLR value between Group 3 and Group 1 (mean difference = - 81.61, p < 0.001), and PLR value between Group 3 and Group 2 (mean difference = - 70.57, p < 0.001). Statistically significant differences were also obtained for the mean value of SII between Group 3 and Group 2 (mean difference = - 1060.60, p = 0.012), the mean value of NLR between Group 3 and Group 2 (mean difference = - 2.24, p = 0.017), and the mean value of NLR between Group 2 and Group 1 (mean difference = - 2.67, p = 0.004).

**Figure 5:**
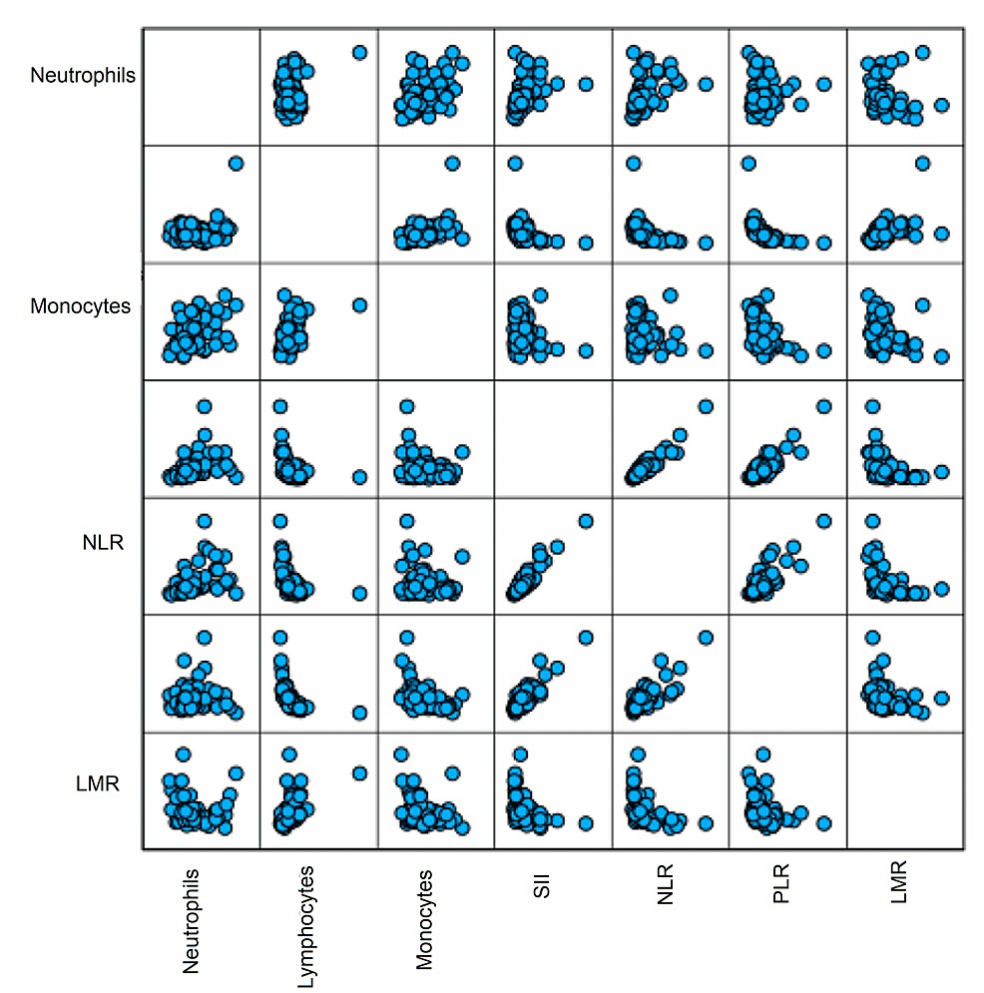
Scatter matrix graph showing statistically significant correlations between the inflammatory markers and some parameters of the CBC found in Group 1. Each square represents one single correlation. SII: systemic immune-inflammation index; NLR: neutrophil-to-lymphocyte ratio; LMR: lymphocyte-to-monocyte ratio; PLR: platelet-to-lymphocyte ratio

Group 1 included some statistically significant correlations (Figure 5). Very good, highly statistically significant correlations were detected between NLR and SII (p < 0.001), PLR and SII (p < 0.001), and between NLR and PLR (p < 0.001). Acceptable correlations are seen between SII and LMR (p = 0.003) and between PLR and LMR (p = 0.015).

## Discussion

In the last decades, many studies have revealed a strong correlation between the immune-inflammatory system perturbation and tumorigenesis, cancer progression, tumor growth, and patient survival [8-10]. Hu et al. proposed for the first time the use of SII as a prognostic parameter reflecting the host immune-inflammatory responses. The chosen cut-off point for the SII in Hu et al.'s study on patients suffering from hepatocellular carcinoma was 330 and his results revealed that patients with SII values over 330 were significantly associated with early recurrence, vascular invasion, and larger tumors [11]. Other studies have revealed that elevated SII values represent a bad prognostic factor for overall survival in lung adenocarcinoma, prostate cancer, SCLC, gastrointestinal tract cancer, hepatocellular carcinoma, and acral melanoma; however, there is no standard cutoff point for SII indicating that in various types of cancer, inflammation plays different roles [12,13]. The study conducted by Li et al. on patients with BM of NSCLC using Kaplan-Meier analysis revealed that higher values of the SII, NLR, and PLR were associated with shorter overall survival [14]. Our results reveal a statistically significant gradual increase in the average SII values between the three groups (p < 0.001). The highest difference in SII values was seen between healthy subjects and patients with LC and BM (Tukey test p < 0.001) in which the mean SII was approximately four times higher compared to healthy subjects (577.41 in Group 3 vs. 2020.98 in Group 1). Patients in the premetastatic stage of the disease had SII values that were almost three times higher compared to healthy subjects (Tukey test p = 0.012) (577.41 in Group 3 vs 1638.01 in Group 2) (Figure 1).

Inflammation is crucial for tumorigenesis and cancer progression. This inflammatory status is represented by an increase in the number of neutrophils and a decrease in the number of lymphocytes. By releasing elevated levels of vascular endothelial growth factor (VEGF) and matrix metalloproteinase (MMP)-9, neutrophils enhance angiogenesis at the tumoral site, promoting cancer development and progression. Also, neutrophils have the capability of deteriorating the extracellular matrix, in this way facilitating tumor cell invasion and also impeding the cytolytic activity of immune cells like lymphocytes [15,16]. Lymphocytes secrete interferon‐gamma (IFN‐γ) and tumor necrosis factor-alpha (TNF-α) which are very important for inducing cytotoxic cell death and inhibiting tumor proliferation and tumor migration [17].

In the meta-analysis published by Wang et al., elevated pretreatment values of the NLR were associated with shorter progression-free survival and overall survival in NSCLC patients [18]. Cho et al. have shown in their study, which was performed on a cohort of 166 patients suffering from NSCLC who were treated with radiosurgery and concomitant immunotherapy or targeted therapy, that patients with pretreatment NLR values of less than 5 had a much longer estimated medial survival that patients with pretreatment NLR values over 5 [19]. Another important result of their study was that each increase of the NLR value with 1 resulted in an increase in death risk by 5.4%. Our findings regarding the average values of NLR (ANOVA test p < 0.001) are in accordance with the above studies: patients in the healthy group exhibited the lowest average NLR value (NLR = 2.34) followed by the patients with LC without metastases whose average NLR value (4.58) has almost doubled (Tukey test p = 0.017) and patients with LC and BM presented the highest NLR average value (NLR = 7.25) indicating a shorter life expectancy (Tukey test p < 0.001). 

Platelets protect the circulating tumor cells from shearing and monocytes have a role in tumor-associated angiogenesis and antitumor response suppression [20]. Starzer et al. identified significantly higher PLR values in patients with simultaneous progressive extracranial disease at brain metastasis diagnosis compared to patients with stable extracranial disease at brain metastasis diagnosis. The study was conducted mostly on patients suffering from LC but also included various types of other cancers like breast cancer, melanoma, and renal cell carcinoma [21]. In our study, the difference between the mean PLR values of the oncological groups is not statistically significant even though we can see a mild increase from 188.42 in patients with stable LC to 199.46 in patients with LC and BM (Tukey test p > 0,05). Gu et al. conducted a meta-analysis that included 3430 patients diagnosed with NSCLC and concluded that elevated values of PLR are associated with poor overall survival, especially in Caucasians, and poor disease-free survival or progression-free survival [22]. The cut-off point used for the PLR in their study was 180. From the results of the current study, we can see that compared to healthy subjects (who presented an average PLR value of 117,84), patients from both oncological groups had average PLR values over the previously reported cut-off point of 180 (ANOVA test p < 0.001), indicating a bad prognosis (Figure 3). 

Li et al. revealed that patients in the higher LMR group had longer overall survival [14]. Our study is in accordance with Li et al.'s study although our results regarding LMR were not statistically significant (p > 0.05). The highest LMR values were seen in healthy subjects (average LMR = 3.56) and patients presenting in the metastatic stage of the disease had the lowest LMR average values (LMR = 2.97) (Tukey test p = 0.198). There was a difference in the average LMR values between healthy subjects and patients from Group 2 (3.56 in Group 3 vs 3.43 in Group 2) but this was not significant (p > 0.05) (Figure 4).

The limitations of this study include the possibility that undiagnosed subclinical infections may have altered the analyzed immune parameters, despite excluding patients with active infections. Additionally, we did not perform a separate analysis on patients with SCLC and NSCLC, instead including them all in the group of patients with LC due to the limited number of SCLC patients. Other limitations are the relatively small number of patients included and the fact that this study was conducted at a single center, which may have limited the generalizability of our findings. 

## Conclusions

This study provides valuable insights into the dynamics of inflammatory indexes in LC patients with and without BM. Following the ANOVA test, highly statistically significant differences in the average values of SII, NLR, and PLR were found, which gradually increased between healthy subjects, patients with LC without BM, and patients with BM of LC origin. Another important observation was the statistically significant increase in the NLR average value between the group of patients with LC without BM and the group of patients with BM of LC origin, as indicated by the post hoc test (Tukey). These gradual increments of the immune-inflammatory indexes from healthy subjects to LC patients with BM underscore their association with disease progression and poorer outcomes.

Our findings emphasize the prognostic significance of elevated SII, NLR, and PLR levels in various cancers. Moreover, our analysis revealed notable correlations between NLR and SII, PLR and SII, and between NLR and PLR within the group of patients with LC without BM, further supporting their role in tumor microenvironment modulation. While LMR did not exhibit significant differences among the groups, its trend toward lower values in metastatic patients warrants further investigation.
